# Ubiquitylation Functions in the Calcium Carbonate Biomineralization in the Extracellular Matrix

**DOI:** 10.1371/journal.pone.0035715

**Published:** 2012-04-25

**Authors:** Dong Fang, Cong Pan, Huijuan Lin, Ya Lin, Guangrui Xu, Guiyou Zhang, Hongzhong Wang, Liping Xie, Rongqing Zhang

**Affiliations:** 1 Institute of Marine Biotechnology, School of Life Sciences, Tsinghua University, Beijing, China; 2 Protein Science Laboratory of the Ministry of Education, Tsinghua University, Beijing, China; RMIT University, Australia

## Abstract

Mollusks shell formation is mediated by matrix proteins and many of these proteins have been identified and characterized. However, the mechanisms of protein control remain unknown. Here, we report the ubiquitylation of matrix proteins in the prismatic layer of the pearl oyster, *Pinctada fucata*. The presence of ubiquitylated proteins in the prismatic layer of the shell was detected with a combination of western blot and immunogold assays. The coupled ubiquitins were separated and identified by Edman degradation and liquid chromatography/mass spectrometry (LC/MS). Antibody injection *in vivo* resulted in large amounts of calcium carbonate randomly accumulating on the surface of the nacreous layer. These ubiquitylated proteins could bind to specific faces of calcite and aragonite, which are the two main mineral components of the shell. In the *in vitro* calcium carbonate crystallization assay, they could reduce the rate of calcium carbonate precipitation and induce the calcite formation. Furthermore, when the attached ubiquitins were removed, the functions of the EDTA-soluble matrix of the prismatic layer were changed. Their potency to inhibit precipitation of calcium carbonate was decreased and their influence on the morphology of calcium carbonate crystals was changed. Taken together, ubiquitylation is involved in shell formation. Although the ubiquitylation is supposed to be involved in every aspect of biophysical processes, our work connected the biomineralization-related proteins and the ubiquitylation mechanism in the extracellular matrix for the first time. This would promote our understanding of the shell biomineralization and the ubiquitylation processes.

## Introduction

A vast array of organisms can precipitate minerals via a process known as biomineralization. To accurately control mineral deposition, biogenic minerals generally have specific attributes that distinguish them from their inorganic counterparts [1]. It is well known that the biomineralization product of the molluscan shell is calcium carbonate. The shell of the pearl oyster, *Pinctada fucata*, consists of two different forms of calcium carbonate, *i.e.*, aragonite in the inner nacreous layer and calcite in the outer prismatic layer [2], [3]. Previous studies have shown that the matrix proteins account for less that 5% (w/w) of the shell, but they are the major component responsible for the control of shell microstructure [4], [5]. Since the discovery of nacrein [6], MSI31 [7], MSI60 [7], and Lustrin A [8], many other matrix proteins have been purified, cloned and characterized [9]–[18]. However, the mechanism that controls these matrix proteins remains largely unknown, because studies at the molecular control level have been limited [19], [20].

Several proteomics and genomics studies have been conducted to understand the process of biomineralization control, where ubiquitin was found to be present in biogenic minerals [21]–[23]. Our previous study also showed that ubiquitin was expressed at key points during larval shell formation [24]. An 8.5 kDa protein that is involved in diatom wall formation was also found to be homologous to ubiquitin [25]. Ubiquitin is highly conserved among eukaryotic organisms, where it functions as a post-translational protein modifier [26], [27]. Ubiquitin can attach to target proteins via lysyl residues and multiubiquitylation occurs with the addition of ubiquitin to several lysines on proteins, whereas polyubiquitylation occurs with the addition of several ubiquitin molecules to a single lysyl residue in a protein [28]. These modifications allow ubiquitin to have an essential role in all aspects of cellular physiology, including protein degradation, receptor trafficking, DNA repair, cell cycle progression, gene transcription, autophagy, and apoptosis [29]–[35]. However, no direct evidence has yet been provided to characterize the role of ubiquitylation in the control of biomineralization in *P. fucata*.

The current study reports the analysis of ubiquitylation in matrix proteins. The presence and function of the ubiquitylated proteins was evaluated by *in vivo* and *in vitro* biochemical analysis. Ubiquitylated matrix proteins repressed the rate of precipitation and induced calcite formation in the presence of magnesium. Our results demonstrate that ubiquitylation participates in the control of calcium carbonate biomineralization in *P. fucata*.

## Results

### Ubiquitylated proteins in the prismatic layer

**Figure 1:**
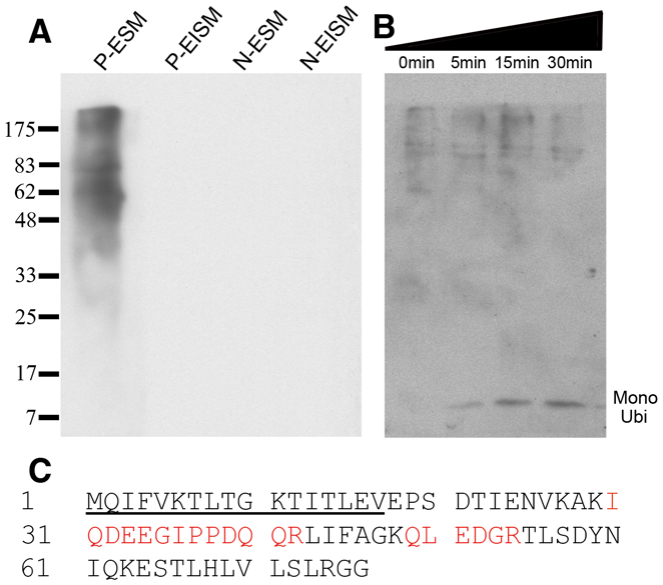
Ubiquitylation of *P. fucata* matrix proteins. (A) The ubiquitylated proteins were characterized by western blotting of EDTA extracts of nacre and prisms separated from the shell. The ubiquitylated proteins were mainly present in the EDTA-soluble matrix of calcitic prisms. P-ESM, EDTA-soluble matrix of the prismatic layer; P-EISM, the EDTA-insoluble matrix of the prismatic layer; N-ESM, EDTA-soluble matrix of the nacreous layer; N-EISM, the denatured fraction of the EDTA-insoluble matrix of the nacreous layer. (B) Time-course reaction of isopeptidase with the EDTA-soluble matrix fraction of the prismatic layer. Reaction products were analyzed by western blotting. The reaction was performed at 37°C with a volume of 15 µL containing 0.1 µM of isopeptidase, 2 µg of substrate, for the indicated times. Mono Ubi, mono-ubiquitin. (C) Amino acid sequence of ubiquitin showing the residues identified by Edman degradation (underlined) and the peptide sequences identified by LC-MS analysis (red highlights).

Studies have shown that ubiquitin may be involved in the processes of biomineralization. Biochemical analyses were carried out to investigate whether ubiquitylated proteins are present in the nacreous layer, the prismatic layer, or both. Monoclonal antibodies raised against mono-ubiquitin and poly-ubiquitin were used for immunodetection in EDTA extracts of separated nacre and prisms. Western blot analysis detected ubiquitylated proteins exclusively in the EDTA-soluble matrix (ESM) of the prismatic layer. The matrix of the nacreous layer and the EDTA-insoluble matrix (EISM) of the prismatic layer lacked ubiquitin-specific signals (Fig. 1A).

To further confirm the presence of ubiquitylated proteins, isopeptidase was used to catalyze the cleavage of an isopeptide bond attaching the terminal diglycine to ubiquitin [36]. A time-course reaction was performed. The anti-ubiquitin signal was stronger in the 8.5 kDa line over time, *i.e.*, the molecular weight of ubiquitin in most animals (Figure 1B). The 8.5 kDa proteins were analyzed by Edman degradation and 17 residues of the N-terminal sequence (MQI FVK TLT GKT ITL EV) were 100% homologous with the ubiquitin found in various mollusks. Based on the N-terminal residues, we cloned the cDNA sequence of ubiquitin using 3′-RACE and 5′-RACE to further analyze whether the 8.5 kDa protein was ubiquitin. This 8.5 kDa protein was further tested by using liquid chromatography/mass spectrometry (LC/MS) (Figure 1C). The LC/MS data confirmed that the 8.5 kDa protein was *P. fucata* ubiquitin.

**Figure 2:**
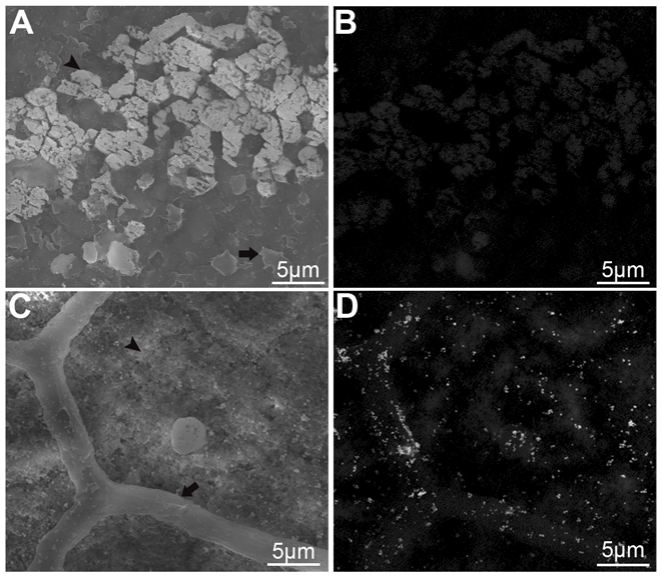
Immunogold labelling of ubiquitylated proteins in the nacreous layer (A and B) and the prismatic layer (C and D). The complexes formed by the antigen–1st antibody–2nd gold-labeled antibody were identified as tiny bright spots by SEM-BSE. (A) SEM image of immunogold staining of the nacreous layer surface. The black arrow indicates the EDTA-insoluble framework, while the black arrowhead indicates the etched aragonitic tablets. (B) SEM-BSE image of the same area shown in (A). (C) SEM image of the immunogold-stained prismatic layer surface. The black arrow indicates the EDTA-insoluble framework, while the black arrowhead indicates the etched prisms. (D) SEM-BSE image of the same area shown in (C). Ubiquitylated proteins were detected in the prisms and on the surface of the EDTA-insoluble framework. Scale bars, 5 µm in (A)–(D).

The EDTA-etched prismatic and nacreous layers were immunogold-labeled using anti-ubiquitin antibodies as the first antibody and 5 nm gold-labeled antibodies as the second antibody to elucidate the microstructural distribution of native ubiquitylated proteins within the shell. The EDTA treatment allowed the calcium carbonate found in the shell to be slightly etched away to expose proteins within the shell's structure. The aragonitic tablets in the nacreous layer were etched away (Figure 2A, black arrowhead), but the intertabular matrix between them was not affected because it is an EDTA-insoluble framework (Figure 2A, black arrow). The nacreous layer was not labeled by gold because there were no bright, tiny spots to indicate high atomic number gold elements in the back-scattered electron mode SEM (SEM-BSE) (Figure 2B). The calcitic prisms were etched away (Figure 2C, black arrowhead) and the insoluble framework was exposed to the antibody (Figure 2C, black arrow) in the prismatic layer. The tiny spots indicated that ubiquitylated proteins were present in the prisms and on the surface of the framework (Figure 2D). Sections were incubated without the anti-ubiquitin antibodies to provide a negative control and no staining was observed in these sections (data not shown). The distribution of the ubiquitylated proteins in the shell microstructure confirmed the results of the western blot analysis.

### In vivo investigation of the role of ubiquitylated proteins during shell mineralization

**Figure 3:**
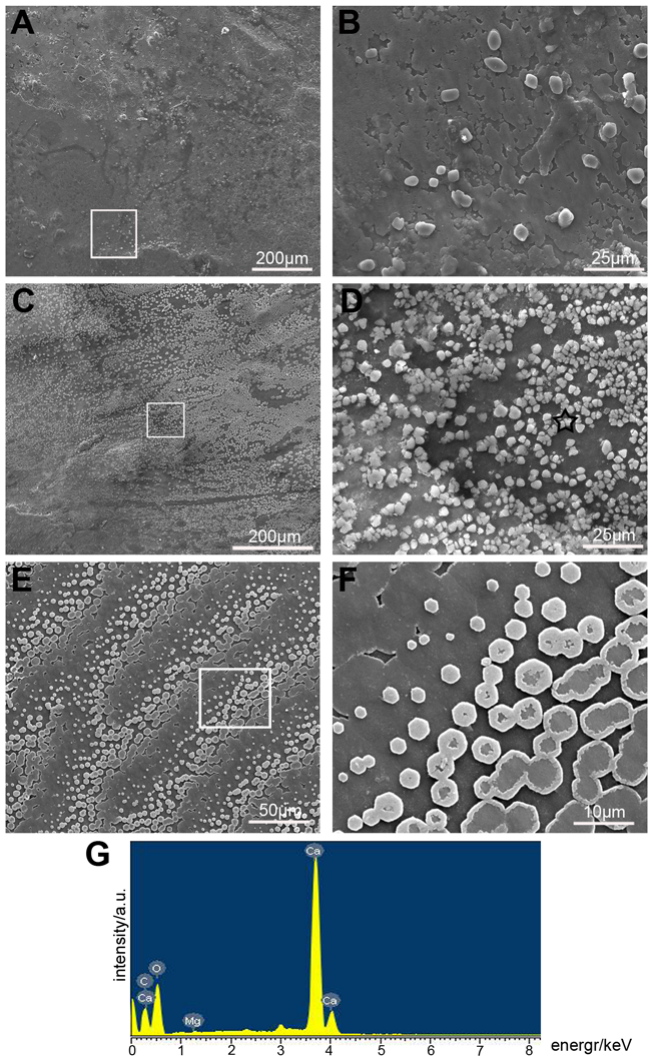
The physiological functions of the ubiquitylated proteins were inhibited by antibody injection. (A) SEM image of the inner surface of the low dosage antibody-injected group. The stair-like growth pattern was disturbed. (B) Enlargement of the box shown in (A), illustrating the crystals deposited on the inner nacreous layer surface. (C) SEM image of the inner surface of the high dosage antibody-injected group. More crystals were randomly accumulated. (D) Enlargement of the box shown in (C), illustrating that the crystals were linked together to form a new layer. (E) SEM image of the inner surface of preimmune rabbit serum injected *P. fucata* shell showing the stair-like growth pattern. (F) Enlargement of the box shown in (E), illustrating the flat tablets. (G) Energy-dispersive x-ray spectroscopy analysis of the deposition (black asterisk) shown in (D). Scale bars, 200 µm in (A) and (C); 50 µm in (E); 25 µm in (B) and (D); 10 µm in (F).

An *in vivo* antibody injection assay was conducted to further clarify the role of ubiquitylated proteins in shell formation. Anti-ubiquitin antibodies were injected into the extrapallial fluid of *P. fucata* at dosages of 0.5 µg or 1 µg of protein per gram wet weight of oysters per day. Three days after the antibodies were injected, the inner surfaces of the nacre were observed by SEM. In the preimmune rabbit serum injected group, the surface was packed with small flat tablets of aragonite with a stair-like growth pattern which was similar to that for untreated samples (Figure 3E and 3F). Compared with the preimmune rabbit serum injected group, small tablets with variable shapes were randomly accumulated on the surface of the nacreous layer in eleven of the fifteen individuals in the low dosage (0.5 µg/g/d) antibodies-injected group (Figure 3A and 3B). These abnormal tablets disturbed the stair-like growth pattern of the inner shell. This abnormal phenomenon was more significant in the high dosage (1 µg/g/d) injected group (Figure 3C and 3D). More crystals were accumulated on the surface and they linked together to form a new layer in twelve of the fifteen tested individuals. Chemical composition analysis by energy-dispersive x-ray spectroscopy showed that the irregular precipitates were composed of carbon, oxygen, calcium, and a small amount of magnesium (Figure 3G). The physiological functions of ubiquitylated proteins were inhibited, which led to the anomalous deposition of calcium carbonate on the surface of the nacreous layer. In addition, the surfaces of the prismatic layer of the antibody injected groups were not affected. These observations suggested that these ubiquitylated proteins may function as a negative regulator of calcium carbonate deposition.

### Immunoaffinity chromatography of the ubiquitylated proteins

**Figure 4:**
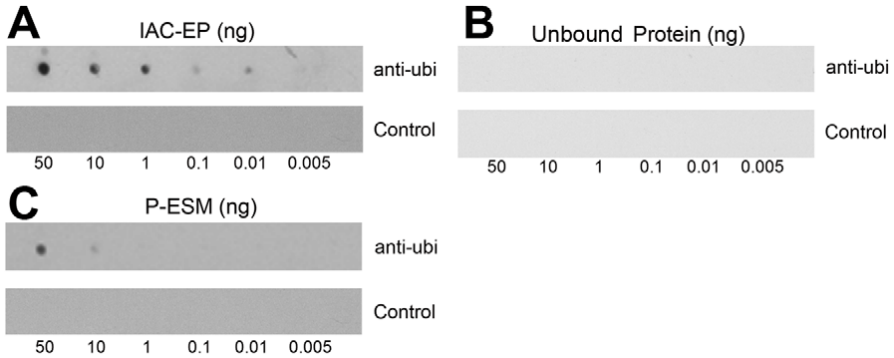
Detection of ubiquitylated proteins in the IAC-enriched proteins. The IAC-enriched proteins (A) and IAC unbound proteins (B) and EDTA-soluble matrix of the prismatic layer (C) were analyzed using a dot blot. The extracts were compared with known amounts of IAC-enriched proteins, which were serially diluted and treated as positive controls. The negative controls were run without the anti-ubiquitin antibodies.

This study found that the ubiquitylated proteins in the ESM of the prismatic layer played an important role in shell formation. The functions of these proteins were further investigated by separating the ubiquitylated proteins by immunoaffinity chromatography (IAC). Anti-ubiquitin antibodies were assembled on a column to purify the ubiquitylated proteins. The purified proteins were then analyzed using a dot blot assay. Figure 4 shows that the ubiquitylated proteins in the purified sample were enriched by at least 1000-fold, while a dot of 0.01 ng enriched proteins was detected in the analysis (Figure 4A). The sensitivity of the substrate in the dot blot assay was approximately 1∼3 picograms according to the manufacturer's instruction (Millipore), and this suggests that the enriched proteins were ubiquitylated proteins of the ESM of the prismatic layer. The immune-affinity column unbound material was also tested by dot blot and the data showed no ubiquitylated proteins in the unbound material (Figure 4B).

### Interactions in the matrices of the different shell layers

**Figure 5:**
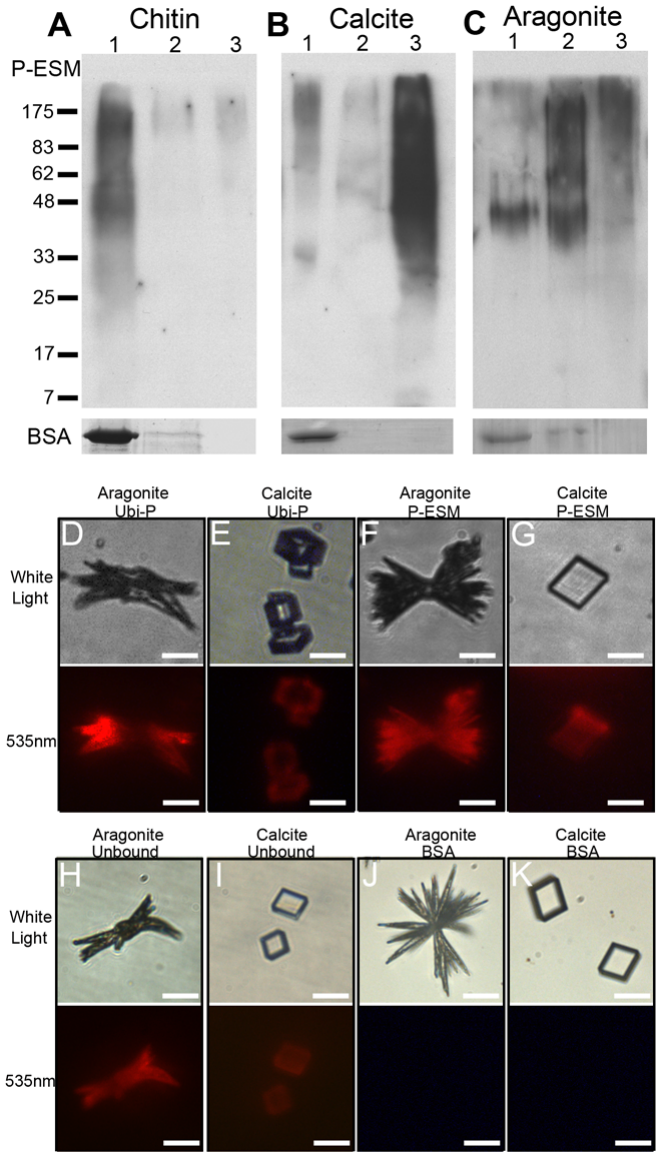
Binding profiles of ubiquitylated proteins. The binding properties of ubiquitylated proteins with chitin (A), calcite (B), and aragonite (C) were tested by western blot. Lane 1, water washings; lane 2, 0.2 M NaCl washings; lane 3, extracted with SDS/β-mercaptoethanol for 10 min at 100°C. Ubiquitylated proteins were completely eluted with water in the chitin-binding assay (A), and with the denaturing solution in both the calcite- and aragonite-binding assays (B and C). BSA was included as a negative control. Ubiquitylated proteins showed different binding patterns with aragonite (D) and calcite (E) in the *in vitro* crystal-protein binding experiment. The EDTA-soluble matrix of the prismatic layer bound to all the surfaces of aragonite (F) and calcite (G). The IAC-unbound proteins bound to all the surfaces of aragonite (H) and calcite (I). BSA was used as a negative control showing no signals with aragonite (J) or calcite (K). Scale bar: 20 µm in (D)–(K).

The main matrix of the *P.fucata* shell is composed of calcium carbonate, which forms calcite in the prismatic layer and aragonite in the nacreous layer, while chitin constitutes the major insoluble component of the organic framework. A number of binding assays were conducted to analyze the binding activity of the ubiquitylated proteins with the major components of the shell. In the chitin-binding assay, the proteins were incubated with chitin and washed with distilled water, saline, and a hot denaturing solution. Ubiquitylated proteins were completely eluted with water showing that the proteins had a very weak binding activity with chitin (Figure 5A). In the calcite-binding assay, the ubiquitylated proteins were not completely eluted with water and saline, whereas they were eluted with the denaturing solution, which indicated that these proteins could bind tightly to calcite (Figure 5B). With aragonite as the substrate, some proteins were eluted with water, the majority of the proteins were eluted with saline, and some proteins of high molecular weight were eluted with Laemmli buffer, which suggests that these proteins could also bind tightly to aragonite (Figure 5C).

These findings suggest that the ubiquitylated proteins could bind to calcite and aragonite. We tested this hypothesis by incubating the rhodamine-labeled ubiquitylated proteins with calcite and aragonite. The binding of ubiquitylated proteins was limited to the needle-like extensions of aragonite and the edges of calcite (Figure 5D and 5E). In contrast, the fluorescence appeared on all the aragonite and calcite surfaces when ESM of the prismatic layer or the immuno-affinity column unbound material was tested (Figure 5F–I). BSA was used in the control experiment and no fluorescent signal was detected on the surface of calcium carbonate crystals (Figure 5J and 5K).

### In vitro effect of ubiquitylated proteins on calcium carbonate crystallization

**Figure 6:**
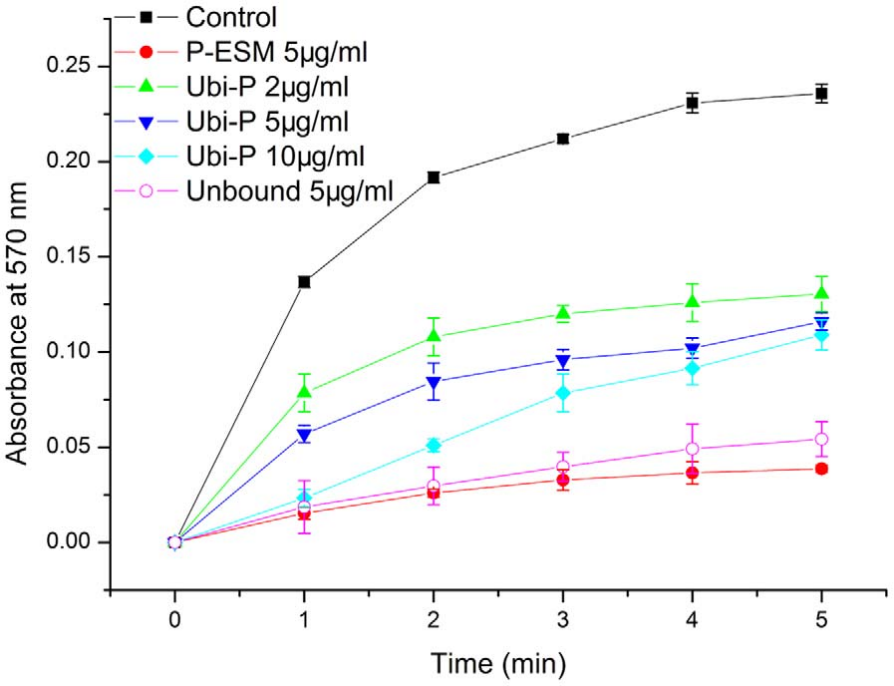
Inhibitory activity of ubiquitylated proteins during calcium carbonate precipitation. Changes in the turbidity of the assayed solutions are shown. ▪ BSA (10 µg/ml) was used as the negative control. • The EDTA-soluble matrix from the prismatic layer (5 µg/ml) was used as the positive control. ▴ 2 µg/ml ubiquitylated proteins. ▾ 5 µg/ml ubiquitylated proteins. ♦ 10 µg/ml ubiquitylated proteins. ○ 5 µg/ml IAC unbound fraction.

A series of calcium carbonate precipitation experiments was performed to analyze the effect of ubiquitylated proteins. The rate of calcium carbonate precipitation was recorded based on the absorbance increase at 570 nm in a saturated calcium carbonate solution. Compared with the control, the addition of ESM of the prismatic layer significantly decreased the rate of precipitation (Figure 6). The same concentration of ubiquitylated proteins decreased the rate of precipitation less compared with the ESM of the prismatic layer. When the immuno-affinity column unbound material was used, the rate of precipitation decreased more than when the ubiquitylated proteins were added to the system.

**Figure 7:**
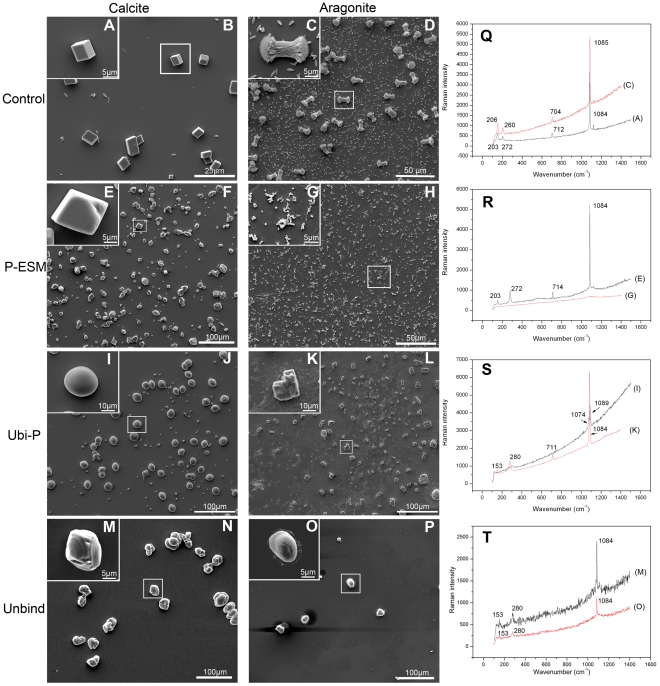
*In vitro* crystallization experiments in the presence of ubiquitylated proteins. The effect of ubiquitylated proteins on the growth of calcium carbonate crystals was tested in two crystallization systems, *i.e.*, calcite growth in the left column and aragonite growth in the middle column. (B) 30 µg/ml BSA was added as the negative control. (F) 30 µg/ml EDTA-soluble matrix of the prismatic layer was added as the positive control. The crystals were etched. (I) 30 µg/ml ubiquitylated proteins were added to the system to induce the formation of vaterite. (N) 30 µg/ml IAC unbound fraction was added. (D), (H), (L), and (P) are the same as those shown in the left column, except that 50 mM Mg^2+^ was added to these systems. (H) No crystals were formed in the aragonite growth systems, when EDTA-soluble matrix of the prismatic layer was added. (L) The ubiquitylated proteins induced calcite formation at a 30 µg/ml concentration. (P) The IAC unbound fraction could induce the formation of round crystals. Right column, Raman spectra of the crystals formed in the left and middle columns. (A), (E), (I), (M), (C), (G), (K), and (O) show enlarged images of the boxed regions in (B), (F), (J), (N), (D), (H), (L), and (P) respectively. Scale bars: 100 µm in (F), (J), (L), (N) and (P); 50 µm in (D) and (H); 25 µm in (B); 10 µm in (I) and (K); 5 µm in (A), (C), (E), (G), (M) and (N).

To evaluate the effect of these ubiquitylated proteins on the growth of calcium carbonate, two individual *in vitro* crystal growth assays were performed to investigate the formation of aragonite and calcite. The effect of ubiquitylated proteins on the growth of calcite was conducted in a magnesium-free system. In the control experiments performed with 30 µg/ml BSA (Figure 7A and 7B), the precipitated crystals exhibited the typical rhombohedra of calcite with smooth surfaces. In the presence of ESM of the prismatic layer at a concentration of 30 µg/ml, the deposited crystals didn't look consistent, but they were etched away on the edges and corners (Figure 7E and 7F). Raman analysis was used to compare the same specific peaks as those observed in the control experiment, which confirmed the calcitic nature of these crystals (Figure 7Q and 7R). In contrast, the crystals had rounded shapes at 30 µg/ml ubiquitylated proteins (Figure 7I and 7J). These deposits were confirmed to be vaterite by Raman analysis (Figure 7S). The immuno-affinity column unbound material could change the morphology of the deposited crystals to form flexible shapes of calcite (Figure 7M, 7N and 7T).

Magnesium (50 mM) was introduced into the aragonite precipitation system to replicate the composition of the extrapallial fluids found in *P. fucata* [16]. In the control experiments, typical large needle-shaped crystals were formed together with a number of small rod-like polyhedral crystals (Figure 7C and 7D). None of these typical calcium carbonate crystals were observed in the presence of ESM of the prismatic layer (30 µg/ml). Aragonite growth was suppressed in this system and there were some gel-like aggregates (Figure 7G and 7H). The Raman spectra showed that these aggregates were not typical crystals, as indicated by the absence of distinct peaks (Figure 7R). Unlike the ESM of the prismatic layer, the crystals deposited in the presence of ubiquitylated proteins were not needle-shaped and instead they were cuboid-like in shape with some etching at the edges and corners (Figure 7K and 7L). These crystals were confirmed as calcite based on their Raman spectra (Figure 7S). The immuno-affinity column unbound material could induce the formation of round shaped calcite (Figure 7O, 7P and 7T). These immuno-affinity column unbound material induced the formation of calcite in the aragonite precipitation system. As aspein was found in the ESM of the prismatic layer and could induce the formation of calcite [9]. The facilitation of calcite formation may be the effect of the aspein-like proteins in the immuno-affinity column unbound material.

### The function of ubiquitin

**Figure 8:**
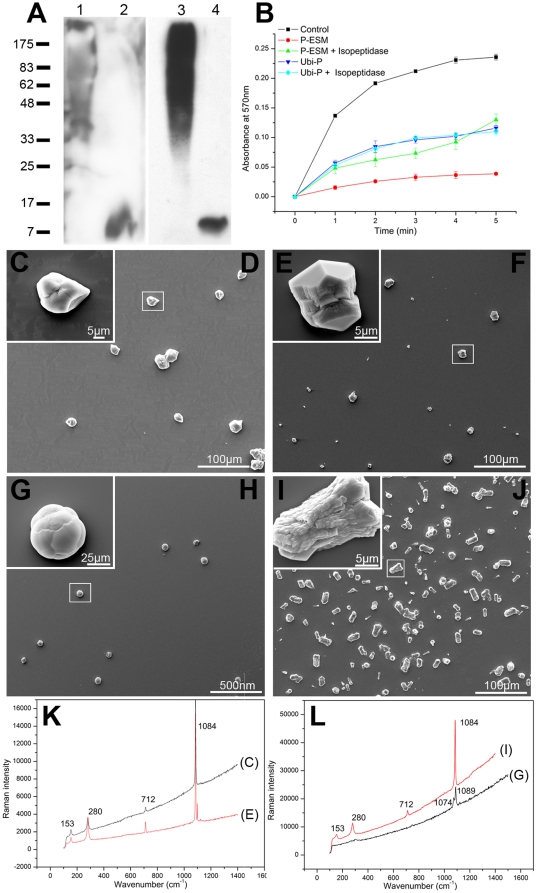
Function of ubiquitins in the ubiquitylated proteins. (A) Catalytic reaction of isopeptidase with ESM of the prismatic layer or ubiquitylated proteins. Different fractions were analyzed by western blotting using anti-ubiquitin antibodies. Lane 1, the ESM of the prismatic layer. Lane 2, the ESM of the prismatic layer after 1 h incubation with isopeptidase. Lane 3, the ubiquitylated proteins. Lane 4, the ubiquitylated proteins after 1 h incubation with isopeptidase. (B) Inhibitory activity of the ubiquitin-removed fraction during calcium carbonate precipitation. ▪ BSA (10 µg/ml) was used as the negative control. • 5 µg/ml ESM of the prismatic layer. ▴ 5 µg/ml isopeptidase incubated ESM of the prismatic layer. ▾ 5 µg/ml the untreated ubiquitylated proteins. ♦ 5 µg/ml isopeptidase incubated ubiquitylated protein. The effects of the isopeptidase incubated ESM of the prismatic layer (30 µg/ml) on crystal growth were tested in a calcite growth system (D) and an aragonite growth system (F). The effects of the isopeptidase incubated ubiquitylated protein (30 µg/ml) on crystal growth were tested in a calcite growth system (H) and an aragonite growth system (J). (C), (E), (G) and (I) show enlargements of the boxes in (D), (F), (H) and (J) respectively. (K) and (L) show the Raman spectra of the crystals formed in (D), (F) (H) and (J). Scale bars: 25 µm in (G); 500 nm in (H); 5 µm in (C), (E) and (I); 100 µm in (D), (F) and (J).

To further evaluate the functions of ubiquitin in these proteins, the ESM of the prismatic layer and the IAC bound ubiquitylated proteins were incubated with isopeptidase to cleave the ubiquitin. After incubation for 1 h, the ubiquitin was completely removed from the proteins (Figure 8A). After the ubiquitin was completely removed from the proteins, SDS-PAGE analysis produced a multi-band result indicating that more than one protein was ubiquitylated (data not shown). The ubiquitin-removal fraction was tested in the subsequent protein function analysis. As shown in the calcium carbonate precipitation experiments, the removal of ubiquitins in the ESM of the prismatic layer could reduce the inhibitory effect of this fraction in calcium carbonate precipitation. However, the ubiquitylated proteins could repress the rate of precipitation to a similar degree with or without ubiquitin (Figure 8B).

In the calcium carbonate growth assay, the removal of ubiquitins from ESM of the prismatic layer could induce the deposition of different shaped calcite without magnesium (Figure 8C and 8D). With magnesium, the removal of ubiquitin could lead to the formation of etched calcite (Figure 8E and 8F) where the ESM of the prismatic layer could induce the formation of some gel-like aggregates (Figure 7G and 7H). The ubiquitin-removed IAC bound ubiquitylated proteins induced the formation of vaterite without magnesium, and calcite with magnesium (Figure 8G–J). With or without magnesium, the presence of ubiquitin or poly-ubiquitin chains (Ub_2–7_, K48-, or K63-linked) in the calcium carbonate crystallization system did not affect crystal deposition or morphology (Figure S1). Thus, the removal of ubiquitin affected the functions of the ESM of the prismatic layer but not the ubiquitylated proteins themselves.

## Discussion

Since the earliest reports of biomineralization [37]–[39], there has been much progress in the analysis of biogenic minerals and this could facilitate applications in the biomaterial and biomedical fields [40]–[42]. It would be useful if we could replicate the natural biomineralization processes. Ubiquitylation is a ubiquitous process that is involved in most biophysical processes. The current study was conducted to advance our understanding of the relationship between biomineralization and ubiquitylation. We focused on the localization and role of ubiquitylated proteins in the shell matrix of *P. fucata*.

Monoclonal anti-ubiquitin antibodies were used to detect ubiquitylated proteins in the shell matrix by western blotting and immunogold-labeling, and this consistently indicated the presence of ubiquitylated proteins in the prisms of the prismatic layer (Figure 1 and 2). However, these data alone did not confirm that these proteins were ubiquitylated. Thus, the ubiquitin gene was cloned for further biochemical analysis. The cloned ubiquitin gene encoded a 76-amino-acid residue protein with a calculated molecular mass of 8.5 kDa. Following the catalytic effect of isopeptidase, the separated 8.5 kDa protein was shown to be ubiquitin by Edman degradation and LC/MS analysis (Figure 1). This result was consistent with an earlier proteomic analysis of biogenic calcium carbonate where ubiquitin peptides were detected [43]. A previous study found that mono-ubiquitin was involved in the silica biomineralization of *Navicula pelliculosa* [44]. However, we detected no mono-ubiquitin in the western blot analysis (Figure 1A). The presence of ubiquitin or poly-ubiquitin chains (Ub_2–7_, K48-, or K63-linked) in the calcium carbonate crystallization system did not affect crystal deposition or morphology (Figure S1). Thus, it was demonstrated that mono-ubiquitin or poly-ubiquitin had little effect on calcium carbonate formation in *P. fucata*. This may be attributable to different control mechanisms in the two species, because *N. pelliculosa* is a type of algae that uses silicon as a biomineral.

We investigated the ubiquitylated proteins by LC/MS analysis. However, the current expression sequence tag (EST) database was not adequate for identifying the proteins [45]–[48]. We are now developing a genomic database to improve the LC/MS assay, which should help to identify the ubiquitylated proteins where lysine is used to form the isopeptide bond.

It is well known that the ubiquitin can be deubiquitylated from its targets and reused in cells [49], [50]. The biomineralization function of the ubiquitylated proteins was tested using a combination of *in vitro* calcium carbonate precipitation and *in vivo* antibody injection assays. The crystal binding profiles of these proteins with calcite were similar to prisilkin-39, which is involved in prismatic layer formation [16], while their binding profiles with aragonite were similar to ACCBP, which stabilizes the crystallization precursor, *i.e.*, amorphous calcium carbonate [51].

The inhibition of crystal formation is also reported in sponge *Clathrina* [52], the ascidian *Pyura pachydermatina* [53] and crayfish *Cherax quadricarinatus* [54], [55]. In the absence of magnesium, these ubiquitylated proteins also inhibited calcite formation to induce the precipitation of vaterite, which can be a precursor of calcite [56]. Given that these proteins were present in prisms and not the prismatic layer framework, some other mediator might be used to induce the formation of calcitic nano-particles in prisms [57]–[60]. The removal of ubiquitin could induce the formation of calcite, which was further supported by *in vivo* observations (Figure 3), where the antibody-mediated perturbation led to the randomly accumulation of crystals on the surface of the nacreous layer. The ubiquitylated proteins act as repressors of magnesium to “knock out” its effect on calcium carbonate formation. Therefore we propose the following hypothesis that the ubiquitylated proteins would reduce the effect of the magnesium on the calcium carbonate crystallization, and then the deposited crystals turned form aragonite to calcite. Relative to calcium, there was a high concentration of magnesium in the extrapallial fluid, which is considered to be the final medium for shell calcification [51], [61], [62]. Crystal nucleation and growth inhibition are both antagonistic mechanisms that are involved in shell formation [63]. Overall, these findings suggest that these ubiquitylated proteins can inhibit the spontaneous crystallization of aragonite and slow down the rate of calcite precipitation to form fine microstructures in the prismatic layer.

It's suggested that the attachment of ubiquitin to a target protein might serve to modulate its activity [49], [64], [65]. The functions of these ubiquitylated proteins didn't seem to be altered with the removal of ubiquitin when only the ubiquitylated proteins were used for the analysis (Figure 8 and S1). However, when ESM of the prismatic layer was tested after enzymatic removal of ubiquitins, the potency of the ESM to inhibit precipitation of calcium carbonate was decreased, and its capacity to influence the morphology of calcium carbonate crystals was changed (Figure 8). Not only one protein is sufficient to induce the formation of the prismatic layer [57]–[60]. Like Pif in shell formation, N16 was needed to achieve the full function of Pif 97 and Pif 80 in shell formation [17]. Simple usage of ubiquitylated proteins in the calcium carbonate growth assay could overlook the function of other proteins in the ESM or the binding of proteins in the ESM to form large protein complex to mediate calcium carbonate crystallization.

In conclusion, the current study demonstrates that ubiquitylation is involved in *P. fucata* shell formation. Further studies on ubiquitylated proteins and how they are ubiquitylated would enhance our understanding of the shell mineralization process. The involvement of ubiquitylation in biomineralization might indicate a more pervasive, flexible and dynamic role for the ubiquitin signal.

## Materials and Methods

### Animals

The oyster, *P. fucata*, was purchased from Guofa Pearl Farm, Beihai, Guangxi Province, China. Animals were maintained in glass aquariums filled with aerated artificial seawater (Sude Instant Sea Salt, 3% at 20°C) for 3 days prior to experimentation.

### Matrix extraction

The preparation of EDTA-soluble and EDTA-insoluble matrix of different layer of shell was performed as described by Kong et al. [16] with some modifications. In the extraction of the soluble matrix, the supernatant was collected by centrifugation at 13,000 rpm for 30 min at 4°C, and then desalted by dialysis and ultra-filtration (Amicon, Ultra-15, 3kD). The amount of the protein was measured by a BCA assay kit (Pierce), according to the manufacturer's instruction.

### Detection of ubiquitylated proteins in shell extracts

The existence of ubiquitylated proteins in extracts of the shell of *P.fucata* or after incubation with isopeptidase was conducted by Western blotting. Protein samples were run on an SDS-PAGE with an equal amount and transferred to PVDF membrane (Millipore). Anti-ubiquitin antibody which could recognize the unconjugated and various forms of conjugated ubiquitin (Merck) was used at dilutions of 1∶2000, and HRP-conjugated goat anti-rabbit secondary antibody (Calbiochem) was used at 1∶10,000. The blot was finally incubated with Luminata Crescendo Western HRP substrate (Millipore) and exposed to the X-ray film for 1 minute. For the dot-blot test, solutions containing the extracts of shell matrix were serially diluted with the same buffer and then vacuum-blotted onto a polyvinylidene difluoride membrane. The membrane was incubated with the anti-ubiquitin antibodies diluted at 1∶2000 and then treated as a normal blot.

### Isopeptidase activity assay

The enzymatic reaction was performed in a 15 µl total volume. Indicated quantities of isopeptidase (0.1 µM, BostonBiochem) were pre-incubated for 15 min at 22°C in 50 mM Tris-HCl, pH 7.2, 10 mM DTT to achieve maximum activity. The substrates were added to the reaction at a concentration of 2 µg, and then further incubated at 37°C for the indicated times.

### Identification of the cDNA sequence of ubiquitin

The removed ubiquitin was electro-transferred from the SDS-PAGE gel to a polyvinylidene difluoride membrane, and its N terminus was sequenced via Edman degradation (Applied Biosystem, Procise 491).

Total RNA was extracted from the larval stages using the SV total RNA isolation kit (Promega), according to the manufacturer's instruction. RNA quantity was assessed by measuring OD260/280 with an Utrospec 3000 UV-visible spectrophotometer (Amersham Biosciences). The integrity of RNA was determined by fractionation on 1.2% formaldehyde denatured agarose gel and staining with ethidium bromide. 3′-RACE and 5′-RACE were performed with a SMART RACE cDNA Amplification kit (Clontech). A degenerate sense primer (5′- TGC ARA THT TYG TNA ARA CNY TNA C-3′) was designed according to N-terminal residues of ubiquitin for 3′-RACE. According to the cDNA sequence acquired from the 3′-RACE, a gene-specific antisense primer (5′-CAA TCT CTG TTG GTC TGG GGG AA -3′) was used for 5′-RACE.

In-gel digestions were performed as described [66]. Liquid chromatography/mass spectrometry (LC/MS) analyses were carried out by using an Agilent 6300 Series system (Agilent Technologies, Santa Clara, CA). The analytical column was a Zorbax 300SB C18 analytical column (150 mm×75 µm, 5 µm). Mobile phase A was 2% acetonitrile and 0.1% formic acid, whereas mobile phase B was 80% acetonitrile and 0.1% formic acid. The gradient was 2%–40% mobile phase B in 75 min, followed by 100% mobile phase B for 5 min. The flow rate was 200 nl/min. The mass spectra were analyzed by Spectrum Mill (Agilent).

### Immunolocalization of the ubiquitylated proteins with Gold Particles

This was performed as described in Marin *et al.* [67], with some modifications. The anti-ubiquitin antibodies (Merck) were used as diluted 1∶1000. The diluted (1∶400) goat anti-rabbit antibodies coupled to 5-nm gold particles (Sigma) were used as the second antibodies. Blank experiments were performed similarly without the first antibody step. The samples were sputter-coated with carbon and analyzed with a FEI Sirion2000 scanning electron microscope in the back scattering electron mode.

### In vivo antibody inhibition assay

The mono-clone anti-ubiquitin antibodies (Merck) were injected into the extrapallial space through the zone of the mantle tissue outside the pallial line at the dosage of 0.5 µg and 1 µg per g of wet weight per day. Five specimens in each group were sacrificed 3 days after antibody injection. 1 µg per g of wet weight per day of preimmune rabbit serum was used as a control. The experiment was repeated three times. The shells were separated, washed with Milli-Q water, and immersed in 5% NaOH for 12 h to remove organic components attached to the inner surface. The shells were thoroughly washed with Milli-Q water, air-dried, sputter-coated with 10-nm-thick carbon, and analyzed by FEI Sirion2000 scanning electron microscope, which was equipped with a Kevex energy dispersive x-ray spectrometer for element analysis of crystals.

### Immunoaffinity chromatography affinity assay

HiTrap NHS-activated HP 1 ml column (GE) was used in this assay. The buffer of antibodies was changed to coupling buffer, 0.2 M NaHCO3, 0.5 M NaCl, pH 8.3, by using HiTrap desalting column (GE), according to the manufacturer's instructions. 0.5 mg antibodies in 1 ml coupling buffer was injected onto the column then incubated for 4 hours at 4°C. The excess active groups were deactivated and the non-specifically bound ligands were washed out according to the manufacturer's instructions. The buffer of EDTA-soluble fraction of the prismatic layer was changed to binding buffer, 20 mM Tris-HCl, 50 mM NaCl, pH 7.4, by using HiTrap desalting column. The EDTA-soluble fraction was loaded onto the binding buffer equilibrated IAC column at a flow-rate of 0.2 ml/min. After successive washing with binding buffer was performed until no material appears in the effluent, the desired fraction was eluted from the column by 0.1 M citrate buffer (pH 3.0). Then, the eluate was immediately adjusted to approximately pH 7.4 with 1 M Tris-HCl buffer (pH 8.0).

### Chitin and calcium carbonate crystals binding assay

Chitin binding assays were done as described by Inoue *et al.* [68] and calcium carbonate crystals binding assay were done as described by Suzuki *et al.* [17] with some modifications. The samples (30 µg each) was incubated with 5 mg of chitin (Wako), calcite (Sigma) or aragonite (synthesized as described by *Yasushi et al.* [69]) that had previously been equilibrated with 0.5% ammonium bicarbonate for 1 hour at 4°C. After removal of the solution by centrifugation, the insoluble mixture was successively washed with 200 µl each of distilled water and 0.2 M NaCl twice. Each washing was desalted by ultrafiltration (Millipore, 3-kDa cutoff) and lyophilized, and freeze-dried proteins were resuspended directly in 15 µl of Laemmli sample buffer. The final insoluble residue was boiled in 30 µl of 2% (w/v) SDS containing 20% (v/v) 2-mercaptoethanol for 10 min, and then the supernatant was separated by centrifugation at 12,000 rpm for 5 min. Each washing or supernatant was subjected to SDS-PAGE on a 12% gel under reducing conditions. After electrophoresis, the gel was stained with Coomassie Brilliant Blue for detection of BSA or Western blotted for detection of ubiquitylated proteins as described above.

### In vitro calcium carbonate crystallization assay

Saturated calcium bicarbonate solution was prepared by following the method of Xu *et al.* [70]. An aragonitic crystallizing solution was obtained by the addition of 50 mM magnesium chloride to the former solution. Crystallization experiments were carried out by adding samples to the freshly prepared crystallization solution on a siliconized glass slide at 20°C. After 24 hours, the crystallization solution was removed and the crystals were characterized by FEI Quanta 200 scanning electron microscope and Raman spectra, which were recorded with an excitation wavelength of 514 nm. The spectra were scanned three times for 20 seconds in the range of 100–1400 cm^−1^ with a Renishaw RM2000 spectrometer.

### Protein labeling with rhodamine and crystal binding experiment

This was performed as described previously [51].

### Inhibition of calcium carbonate precipitation assay

The effect of ubiquitylated proteins on calcium carbonate precipitation was examined according to the method of Suzuki *et al.* [15] with some modifications. Sample solution (10 µl) was mixed with 100 µl of 50 mM sodium bicarbonate, pH 8.5. After the addition of 100 µl of 50 mM calcium chloride to the mixed solution, the formation of calcium carbonate precipitates was monitored by recording the changes in the turbidity every minute for 5 minutes by the absorbance at 570 nm using a spectrophotometer (Biorad 680).

## Supporting Information

Figure S1 ***In vitro*** **crystallization experiments in the presence of ubiquitins.** The effect was tested in two crystallization systems, *i.e.*, calcite growth in the left column and aragonite growth in the right column. When (B) 30 µg mL^−1^ ubiquitin (BostonBiochem, human recombinant), (F) 30 µg mL^−1^ K48-linked Ub_2–7_, or (J) 30 µg mL^−1^ K63-linked Ub_2–7_ was added to the system, the crystals showed the normal morphology as those without proteins. (D), (H), and (L) are the same as those shown in the left column, except that 50 mM Mg^2+^ was added to these systems. The crystals showed the morphology as those without proteins too. (A), (E), (I), (C), (G), and (K) show enlarged images of the boxed regions in (B), (F), (J), (D), (H), and (L) respectively. Scale bars: 50 µm in (B), (D); (F), (H), (J) and (L); 5 µm in (A), (C), (E), (G), (I) and (K). (M) Inhibitory activity of ubiquitylated proteins during calcium carbonate precipitation. Changes in the turbidity of the assayed solutions are shown. ▪ BSA (10 µg mL^−1^) was used as the negative control. • Human recombinant ubiquitin (10 µg mL^−1^). ▴ 10 µg mL^−1^ K48 linked ubiquitin_2–7_. ▾ 10 µg mL^−1^ K63 linked ubiquitin_2–7_. Ubiquitin has little effect on the rate of calcium carbonate precipitation.
(PDF)
