# Supplementary figures and images for: Ubiquitylation Functions in the Calcium Carbonate Biomineralization in the Extracellular Matrix
Source: PLoS One. 2012 Apr 25;7(4):e35715. doi: 10.1371/journal.pone.0035715 (PMC3338455; doi:10.1371/journal.pone.0035715)

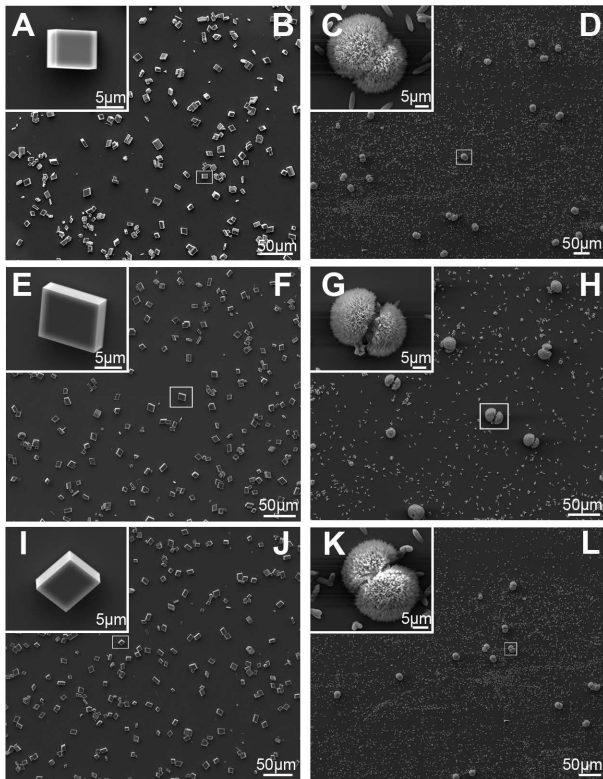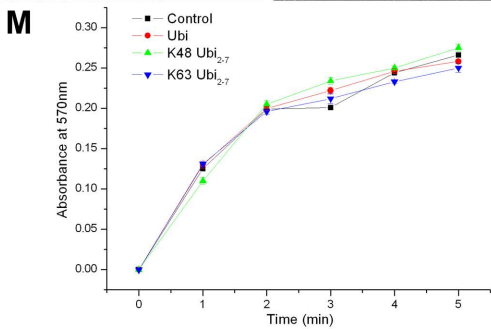

Supplement: Figure S1 — In vitro crystallization experiments in the presence of ubiquitins. The effect was tested in two crystallization systems, i.e., calcite growth in the left column and aragonite growth in the right column. When (B) 30 µg mL−1 ubiquitin (BostonBiochem, human recombinant), (F) 30 µg mL−1 K48-linked Ub2–7, or (J) 30 µg mL−1 K63-linked Ub2–7 was added to the system, the crystals showed the normal morphology as those without proteins. (D), (H), and (L) are the same as those shown in the left column, except that 50 mM Mg2+ was added to these systems. The crystals showed the morphology as those without proteins too. (A), (E), (I), (C), (G), and (K) show enlarged images of the boxed regions in (B), (F), (J), (D), (H), and (L) respectively. Scale bars: 50 µm in (B), (D); (F), (H), (J) and (L); 5 µm in (A), (C), (E), (G), (I) and (K). (M) Inhibitory activity of ubiquitylated proteins during calcium carbonate precipitation. Changes in the turbidity of the assayed solutions are shown. ▪ BSA (10 µg mL−1) was used as the negative control. • Human recombinant ubiquitin (10 µg mL−1). ▴ 10 µg mL−1 K48 linked ubiquitin2–7. ▾ 10 µg mL−1 K63 linked ubiquitin2–7. Ubiquitin has little effect on the rate of calcium carbonate precipitation. (PDF) [file pone.0035715.s001.pdf]
